# Conservative Management of Symptomatic Rathke Cleft Cyst With Recurrent Inflammatory Remission

**DOI:** 10.1210/jcemcr/luaf008

**Published:** 2025-02-10

**Authors:** Masashi Hasebe, Ryo Tsukaguchi, Kimitaka Shibue, Akihiro Hamasaki

**Affiliations:** Department of Diabetes, Endocrinology and Nutrition, Kyoto University Graduate School of Medicine, Kyoto 606-8507, Japan; Department of Diabetes and Endocrinology, Medical Research Institute KITANO HOSPITAL, PIIF Tazuke-Kofukai, Osaka 530-8480, Japan; Department of Diabetes and Endocrinology, Medical Research Institute KITANO HOSPITAL, PIIF Tazuke-Kofukai, Osaka 530-8480, Japan; Department of Diabetes and Endocrinology, Medical Research Institute KITANO HOSPITAL, PIIF Tazuke-Kofukai, Osaka 530-8480, Japan; Department of Diabetes and Endocrinology, Medical Research Institute KITANO HOSPITAL, PIIF Tazuke-Kofukai, Osaka 530-8480, Japan

**Keywords:** arginine vasopressin deficiency, Rathke cleft cyst

## Abstract

Rathke cleft cyst (RCC) is a benign, nonneoplastic cystic lesion arising from embryonic remnants of the Rathke pouch and is increasingly detected with the widespread use of brain magnetic resonance imaging (MRI). Most RCCs remain asymptomatic; however, some present with symptoms such as headaches, visual disturbances, or pituitary dysfunction, often attributed to inflammatory processes within the cyst wall. We report the case of a 68-year-old woman with a year-long history of intermittent headaches, polydipsia, and polyuria. Initial MRI revealed a cystic lesion in the sellar region, and endocrinological assessment confirmed arginine vasopressin deficiency. A serial MRI follow-up revealed recurrent, short-term fluctuations in cyst size, suggesting intermittent inflammatory activity within the RCC. Despite the option for surgical intervention, conservative management alone resulted in the resolution of her headaches and substantial cyst shrinkage. This case underscores the potential for conservative management in symptomatic RCCs with inflammatory features, where hormone replacement and careful imaging follow-up may suffice. It also highlights the need for individualized care and long-term monitoring, especially in cases with dynamic inflammatory behavior.

## Introduction

Rathke cleft cyst (RCC) is a benign cystic lesion derived from embryonic remnants of the Rathke pouch, typically located in the sellar or suprasellar region between the anterior and posterior pituitary lobes. Advances in neuroimaging have led to increased detection rates, making RCC one of the most common nonadenomatous sellar masses [1]. Most RCCs are asymptomatic and incidentally discovered on imaging, where conservative management is generally appropriate [2]. However, in some cases, patients present with symptoms such as headaches or pituitary dysfunction, often attributed to inflammatory changes within the cyst wall [3]. The optimal management approach for these symptomatic cases remains uncertain.

We report the case of a 68-year-old woman with a year-long history of intermittent headaches and polyuria. Initial magnetic resonance imaging (MRI) revealed a cystic sellar lesion and endocrinological assessment confirmed arginine vasopressin deficiency (AVP-D). Close follow-up imaging demonstrated recurrent, short-term fluctuations in cyst size, suggestive of a spontaneous inflammatory process within the RCC. Ultimately, conservative management alone led to the resolution of her headaches and substantial cyst shrinkage. This case highlights the importance of careful imaging follow-up in inflammatory RCC, offering valuable insights into the management of symptomatic RCCs with dynamic inflammatory features.

## Case Presentation

A 68-year-old Japanese woman (height 153.8 cm, weight 50.2 kg, body mass index 21.2) presented to our hospital with a 1-year history of insidious-onset, intermittent frontal headaches, polydipsia, and polyuria. Her urine volume was around 3000 to 4000 mL/day, corresponding to approximately 60 to 80 mL/kg/day. Her medical history included dyslipidemia, managed with a statin, and panic disorder, for which she was taking sulpiride, a dopamine receptor antagonist. She had no other medications or underlying conditions relevant to her presenting symptoms. At assessment, she reported headaches without visual disturbances and had been taking nonsteroidal anti-inflammatory drugs (NSAIDs) as needed, up to 2 to 3 times per day during symptomatic periods. Laboratory tests revealed urinary osmolality of 134 mOsm/kg H_2_O (134 mmol/kg, reference range, 50-1300 mOsm/kg H_2_O [50-1300 mmol/kg]), near the lower end of the reference range, and urinary specific gravity of 1.004 (reference range, 1.005-1.030), which was below normal. Concurrently, serum osmolality was 289 mOsm/kg H_2_O (289 mmol/kg, reference range, 275-290 mOsm/kg H_2_O [275-290 mmol/kg]). These findings—low urinary specific gravity, urinary osmolality near the lower end of the range, and a near-upper-limit serum osmolality—indicated reduced urinary concentrating ability and raised suspicion for AVP-D, prompting further investigation with a hypertonic saline (5%) infusion test and a vasopressin challenge test. The hypertonic saline test demonstrated a markedly blunted AVP response, with plasma AVP levels remaining at less than or equal to 0.5 pg/mL (≤0.41 pmol/L, reference range, < 2.8 pg/mL [2.28 pmol/L]) despite an increase in serum sodium from 145 mEq/L (145 mmol/L, reference range, 138-145 mEq/L [138-145 mmol/L]) to 156 mEq/L (156 mmol/L) (Table 1). In contrast, urine osmolality increased from 208 mOsm/kg H_2_O (208 mmol/kg) to 305 mOsm/kg H_2_O (305 mmol/kg) following subcutaneous administration of 5 units of vasopressin (Table 2). Based on these results, AVP-D was diagnosed as the underlying cause of her polydipsia and polyuria, and further evaluation was initiated to determine its etiology.

**Table 1.** Results of 5% hypertonic saline infusion test

Biomarker	Standard reference range for baseline values	0 min	30 min	60 min	90 min	120 min
Serum osmolality	275-290 mOsm/kg H_2_O (275-290 mmol/kg)	288.0 mOsm/kg H_2_O (288.0 mmol/kg)	297.0 mOsm/kg H_2_O (297.0 mmol/kg)	299.0 mOsm/kg H_2_O (299.0 mmol/kg)	304.0 mOsm/kg H_2_O (304.0 mmol/kg)	307.0 mOsm/kg H_2_O (307.0 mmol/kg)
Serum sodium	138-145 mEq/L (138-145 mmol/L)	145.0 mEq/L (145.0 mmol/L)	148.0 mEq/L (148.0 mmol/L)	151.0 mEq/L (151.0 mmol/L)	154.0 mEq/L (154.0 mmol/L)	156.0 mEq/L (156.0 mmol/L)
Plasma arginine vasopressin	<2.8 pg/mL (<2.28 pmol/L)	<0.4 pg/mL (<0.32 pmol/L)	0.4 pg/mL (0.32 pmol/L)	0.5 pg/mL (0.41 pmol/L)	<0.4 pg/mL (<0.32 pmol/L)	0.4 pg/mL (0.32 pmol/L)

**Table 2.** Results of arginine vasopressin challenge test

Biomarker	Standard reference range for baseline values	0 min	60 min
Serum osmolality	275-290 mOsm/kg H_2_O (275-290 mmol/kg)	302.0 mOsm/kg H_2_O (302.0 mmol/kg)	303.0 mOsm/kg H_2_O (303.0 mmol/kg)
Urine osmolality	50-1300 mOsm/kg H_2_O (50-1300 mmol/kg)	208.0 mOsm/kg H_2_O (208.0 mmol/kg)	355.0 mOsm/kg H_2_O (355.0 mmol/kg)

## Diagnostic Assessment

**Figure 1.**
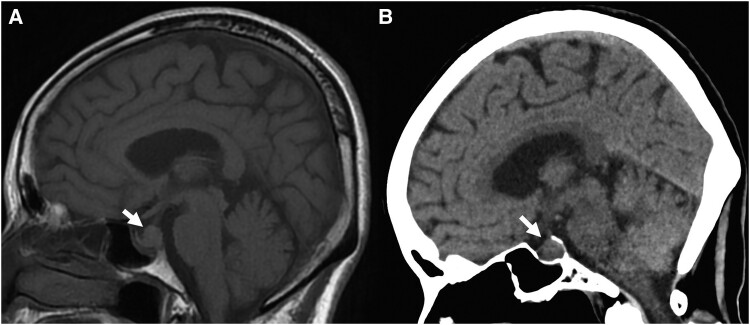
Sagittal images of the head on A, magnetic resonance imaging and B, computed tomography at the initial presentation, showing an enlarged pituitary gland with the absence of the posterior pituitary bright spot and no calcification (arrows).

**Figure 2.**
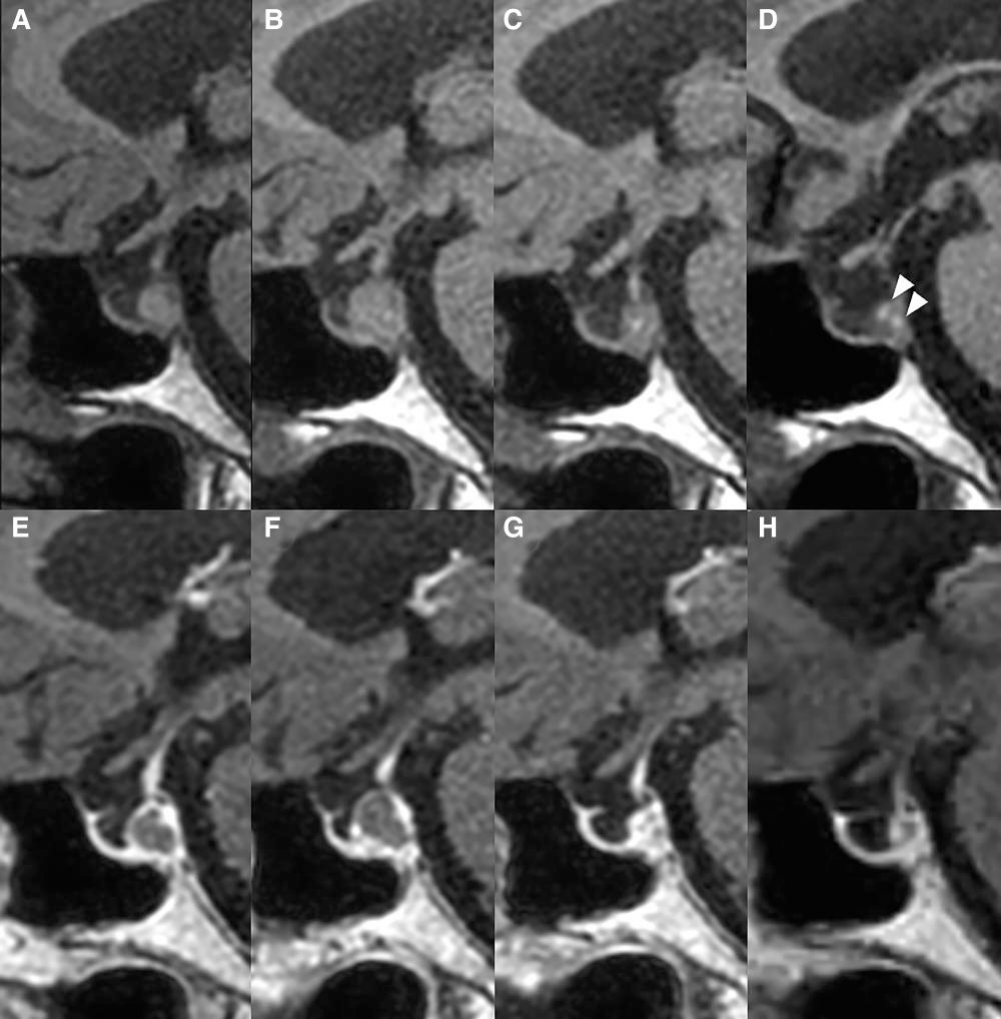
Chronological changes in Rathke cleft cyst (RCC) on T1-weighted magnetic resonance imaging with and without gadolinium enhancement. One week after the initial presentation, spontaneous shrinkage of the RCC was observed along with resolution of headaches (A: noncontrast; E: contrast-enhanced). Three months after the initial presentation, the RCC reenlarged with a recurrence of episodic headaches (B: noncontrast; F: contrast-enhanced). By 6 months, the RCC had reduced in size once again (C: noncontrast; G: contrast-enhanced), and at 9 months, the RCC had further decreased, with a faint reappearance of the posterior bright spot indicated by arrowheads (D: noncontrast; H: contrast-enhanced); however, arginine vasopressin deficiency persisted.

Initial noncontrast MRI revealed an enlarged pituitary gland with an isointense signal and the absence of the posterior pituitary bright spot on T1-weighted imaging (Fig. 1A). No pituitary stalk enlargement was observed, and computed tomography showed no calcification in the sellar region (Fig. 1B). One week later, a follow-up MRI demonstrated a reduction in pituitary size without gadolinium enhancement, suggesting a cystic lesion (Fig. 2A and 2E). At this time, the patient’s headaches had resolved. The acute decrease in cyst size, combined with headache resolution, neurohypophyseal dysfunction, and the isointensity on T1-weighted images, suggested an inflammatory RCC with spontaneous remission [3, 4]. Differential diagnoses included craniopharyngioma and pituitary adenoma (PA); however, craniopharyngiomas typically exhibit calcification and may present with neurological or psychiatric symptoms, while PAs rarely cause AVP-D, making RCC the most likely diagnosis [5]. Additionally, neither craniopharyngiomas nor PAs generally show rapid size changes, supporting an inflammatory origin for RCC in this case.

The anterior pituitary function was assessed through baseline hormone measurements and stimulation tests using gonadotropin-releasing hormone (0.1 mg), thyrotropin-releasing hormone (0.2 mg), corticotropin-releasing hormone (100 μg), and growth hormone-releasing peptide-2 (100 μg) (Tables 3 and 4). Baseline prolactin levels were elevated, likely due to hyperprolactinemia induced by the sulpiride prescribed for panic disorder. This hyperprolactinemia was considered to be associated with lower-than-expected baseline gonadotropin levels for a postmenopausal woman. In contrast, other anterior pituitary hormones and their responses to stimulation were within normal limits. Target gland hormone levels, including free thyroxine (0.83 ng/dL [10.7 pmol/L], reference range, 0.70-1.48 ng/dL [9.0-19.1 pmol/L]) and insulin-like growth factor 1 (61 ng/mL [8.0 nmol/L], reference range for a 68-year-old woman: 60-180 ng/mL [7.8-23.5 nmol/L]), were also within normal limits, suggesting preserved anterior pituitary function apart from the gonadotropin suppression likely caused by hyperprolactinemia secondary to sulpiride.

**Table 3.** Results of gonadotropin-releasing hormone, thyrotropin-releasing hormone, and corticotropin-releasing hormone stimulation test

Biomarker	Standard reference range for baseline values	0 min	30 min	60 min	90 min	120 min
Serum thyrotropin	0.6-4.2 μIU/mL (0.6-4.2 mIU/L)	0.7 μIU/mL (0.7 mIU/L)	6.2 μIU/mL (6.2 mIU/L)	4.5 μIU/mL (4.5 mIU/L)	3.2 μIU/mL (3.2 mIU/L)	2.6 μIU/mL (2.6 mIU/L)
Plasma adrenocorticotropin	7.4-55.7 pg/mL (1.6-12.3 pmol/L)	6.8 pg/mL (1.5 pmol/L)	70.5 pg/mL (15.5 pmol/L)	37.9 pg/mL (8.3 pmol/L)	23.8 pg/mL (5.2 pmol/L)	17.4 pg/mL (3.8 pmol/L)
Serum cortisol	3.7-19.4 μg/dL (102.1-527.0 nmol/L)	12.7 μg/dL (46.9 nmol/L)	24.0 μg/dL (662.2 nmol/L)	25.2 μg/dL (695.3 nmol/L)	23.8 μg/dL (656.6 nmol/L)	25.6 μg/dL (706.3 nmol/L)
Serum luteinizing hormone	11-50 mIU/mL (11-50 IU/L)*^a^*	2.79 mIU/mL (2.79 IU/L)	13.0 mIU/mL (13.0 IU/L)	15.8 mIU/mL (15.8 IU/L)	16.9 mIU/mL (16.9 IU/L)	18.6 mIU/mL (18.6 IU/L)
Serum follicle-stimulating hormone	26-120 mIU/mL (26-120 IU/L)*^a^*	13.5 mIU/mL (13.5 IU/L)	19.6 mIU/mL (19.6 IU/L)	22.8 mIU/mL (22.8 IU/L)	25.1 mIU/mL (25.1 IU/L)	28.3 mIU/mL (28.3 IU/L)
Serum prolactin	3.5-32.7 ng/mL (3.5-32.7 μg/L)	39.5 ng/mL (39.5 μg/L)	81.6 ng/mL (81.6 μg/L)	56.8 ng/mL (56.8 μg/L)	47.5 ng/mL (47.5 μg/L)	45.5 ng/mL (45.5 μg/L)

^*a*^Reference range for postmenopausal women.

**Table 4.** Results of growth hormone-releasing peptide-2 stimulation test

Biomarker	Standard reference range for baseline values	0 min	15 min	30 min	45 min	60 min
Serum growth hormone	0.1-9.9 ng/mL (0.1-9.9 μg/L)	0.7 ng/mL (0.7 μg/L)	8.9 ng/mL (8.9 μg/L)	6.0 ng/mL (6.0 μg/L)	3.7 ng/mL (3.7 μg/L)	2.7 ng/mL (2.7 μg/L)

Although we suggested a transsphenoidal pituitary biopsy for definitive diagnosis, the patient declined due to the invasiveness of the procedure. We, therefore, proceeded with conservative management, involving close monitoring with serial hormonal and MRI evaluations.

## Treatment

Treatment was initiated with oral desmopressin acetate (60 μg twice daily). Following this, her daily urine output decreased to less than 2000 mL, and her polydipsia improved significantly. Surgical intervention was not pursued, as her headaches had resolved. This approach was guided by existing evidence that, while surgery may alleviate headaches associated with inflammatory RCCs, it typically does not reverse endocrine dysfunction [3, 6].

## Outcome and Follow-up

Three months after the initial presentation, the patient experienced a recurrence of intermittent headaches, and a follow-up MRI revealed reenlargement of the RCC (Fig. 2B and 2F), suggesting recurrent inflammation. Given her preference to avoid surgery, conservative management was continued, and the headaches were again treated with as-needed NSAIDs. Three months later, her headaches had resolved again, and the MRI showed a marked reduction in cyst size, indicating re-remission of inflammation (Fig. 2C and 2G). Further cyst shrinkage was observed at an additional 3-month follow-up, and the patient remained headache-free (Fig. 2D and 2H). Notably, a faint posterior bright spot reappeared (Fig. 2D, arrowheads), although AVP-D persisted clinically.

## Discussion

RCC is a nonneoplastic cystic lesion originating from embryonic remnants of the Rathke pouch, with its clinical significance rising as detection rates increase due to the widespread use of brain MRI. Although most RCCs remain asymptomatic, some can become symptomatic, leading to clinical manifestations such as headaches, visual disturbances, and hypopituitarism.

In sellar and suprasellar lesions, including PAs, symptoms like headaches, visual disturbances, and pituitary dysfunction often result from tumor compression of the normal pituitary gland or optic nerves. In contrast, RCC-related symptoms are thought to arise primarily from chronic inflammation around the cyst wall rather than from simple compression by the cyst itself [7]. This inflammatory response to the mucinous content of RCCs can extend to surrounding tissues, as mucin strongly stimulates inflammatory reactions. Consequently, unlike PAs, the size of RCCs does not typically correlate with symptoms such as headaches or hypopituitarism [8]. This pattern was evident in the present case, where, despite the relatively small cyst size, the patient presented with headaches and AVP-D.

In our case, we pursued conservative management, and thus, histological confirmation of cyst wall inflammation was not obtained. However, previous studies report that RCCs with high-intensity or isointense contents on T1-weighted images are more frequently associated with headaches and pituitary dysfunction than those with low-intensity contents, highlighting the utility of MRI in assessing RCC inflammation [3].

For asymptomatic RCCs, typically discovered incidentally, conservative management is generally appropriate, as these cysts remain stable over long-term follow-up and rarely cause pituitary dysfunction [2, 9]. Conversely, surgical intervention may be considered for symptomatic RCCs; however, as both cyst wall inflammation and compression can contribute to symptoms and pituitary dysfunction, surgical indications should be carefully evaluated. In a retrospective study of 72 symptomatic RCC cases, 12.5% experienced postoperative recurrence, while 65% showed no improvement in pituitary function, with particularly low recovery rates for AVP-D [9]. Another study involving 46 RCC cases, of which 33 underwent surgery, reported high rates of headache resolution postoperatively but minimal improvement in pituitary dysfunction [8]. Additionally, a study of 5 RCC cases presenting with AVP-D as the initial symptom found that surgical intervention partially improved AVP-D in cases of acute onset but had no effect on chronic AVP-D [6]. Histological analysis in chronic cases revealed inflammatory cell infiltration into both the anterior and posterior pituitary lobes, with thickened fibrosis beneath the cyst wall, suggesting that prolonged inflammation may lead to irreversible structural changes, with the posterior pituitary particularly vulnerable [6]. In our case, the patient's headaches and cyst size subsided naturally, likely corresponding to intermittent inflammatory responses. Given the prolonged duration of symptoms prior to presentation, surgical improvement of AVP-D was considered unlikely, so a conservative approach was chosen. However, without biopsy or surgery, the possibility of other pathologies cannot be entirely excluded. Even if RCC is confirmed, vigilance for recurrent inflammation remains essential, as this could eventually lead to anterior pituitary dysfunction. Careful, ongoing monitoring with serial hormonal assessments and neuroimaging is therefore warranted. In cases of severe recurrent inflammation, surgical intervention or supraphysiological glucocorticoid treatment may be beneficial [4, 8].

A limitation of our report is the reliance on plasma AVP measurements during the hypertonic saline infusion test for diagnosing AVP-D. Although historically used, these measurements are unreliable due to variability and instability. More recently, copeptin, a stable surrogate marker for AVP, has been shown to provide greater diagnostic reliability [10]. However, in Japan, copeptin measurement is not currently covered by insurance and is not included in the diagnostic guidelines of the Japan Endocrine Society. Incorporating copeptin assays into clinical practice in the future could improve the accuracy of AVP-D diagnosis, particularly in similar clinical scenarios.

This case demonstrates that RCCs can manifest with symptoms such as headaches and endocrine dysfunction driven by inflammatory processes, which may remit spontaneously without surgical intervention. Our findings suggest that, for patients with inflammatory RCC, conservative management with hormone replacement, careful evaluation, and long-term monitoring can be an effective approach. This strategy enables symptom control while avoiding invasive procedures, underscoring the importance of individualized care and close follow-up in managing RCCs with fluctuating inflammatory activity.

## Learning Points

- RCCs can cause symptoms like headaches and pituitary dysfunction due to inflammatory processes rather than simple compression.
- The inflammatory symptoms associated with RCC may naturally remit and recur without surgical intervention, as observed in this case.
- High-intensity or isointensity on T1-weighted MRI may indicate a higher risk of symptomatic inflammation in RCCs.
- Conservative management with appropriate hormone replacement therapy and close monitoring can be effective for symptomatic RCCs with fluctuating inflammatory activity.
- Long-term follow-up with serial imaging and endocrinological evaluation is essential to detect potential recurrence and prevent additional pituitary dysfunction.

## Contributors

All authors made individual contributions to authorship; M.H. was involved in the diagnosis and management of the patient and drafted the manuscript. R.T., K.S., and A.H. provided critical revisions and intellectual content. All authors reviewed and approved the final draft.

## Data Availability

Data sharing is not applicable to this article as no data sets were generated or analyzed during the present study.

## Abbreviations

AVP-D: arginine vasopressin deficiency
MRI: magnetic resonance imaging
NSAIDs: nonsteroidal anti-inflammatory drugs
PA: pituitary adenoma
RCC: Rathke cleft cyst
